# Screening For Occult Heart Failure in Type 2 Diabetes Mellitus Using NT-proBNP: Real-World Evidence From a Tertiary Care Center in India

**DOI:** 10.7759/cureus.72576

**Published:** 2024-10-28

**Authors:** Ameya Joshi, Dhaval Dalal, Sandeep Patil, Harminder Singh, Apoorva Hajirnis, Chandani Seth, Abhijit P Pakhare, Nitinkumar Abdagire, Priti Khatu

**Affiliations:** 1 Endocrinology, Bhaktivedanta Hospital and Research Institute, Thane, IND; 2 Internal Medicine, Bhaktivedanta Hospital and Research Institute, Thane, IND; 3 Cardiology, Bhaktivedanta Hospital And Research Institute, Thane, IND; 4 Cardiology, Bhaktivedanta Hospital and Research Institute, Thane, IND; 5 Community and Family Medicine, All India Institute of Medical Sciences, Bhopal, Bhopal, IND; 6 Pathology, Bhaktivedanta Hospital and Research Institute, Thane, IND

**Keywords:** biomarker, diabetes mellitus, heart failure, nt-probnp, screening

## Abstract

Objective

Heart failure (HF) is an important underrecognized complication of type 2 diabetes mellitus (T2DM). Recent literature and recommendations support screening for HF among T2DM people attending the outpatient department (OPD) in non-emergency settings using a biomarker.

The present study is a retrospective cross-sectional study that assesses the prevalence of screen positivity (S+) for undiagnosed HF among T2DM people (with normal electrocardiogram (ECG) and no history of heart disease) attending the OPD at a tertiary care center in India using N-terminal pro-B-type natriuretic peptide (NT-proBNP). It also highlights the risk factors for S+ for HF.

Methods

This is a retrospective cross-sectional study of the practice of NT-proBNP screening in T2DM to diagnose stage B HF. A total of 1,049 consecutive people with T2DM (age range: 18-75 years) attending the OPD of a tertiary care institute in India were screened for HF using NT-proBNP (cut off S+ >125 pg/mL). Demographic variables, vitals, smoking status, family history, status of hypertension, medications for diabetes, and glycemic control were recorded and correlated with the risk of S+ for HF.

Results

Of the 1,049 people with T2DM, 336 (32.03%) had S+ for HF. Those with S+ had higher age (62.5+9.3 vs 54.2 +10.6 years), longer duration of T2DM (14.4 +7.8 vs 9.6 +6.1 years), positive history for smoking (94 [28%] vs 55 [7.7%]) and tobacco chewing (66 [19.6%] vs 24 [3.4%]), higher blood pressures (both systolic [152.1+19.9 vs 134.6 +15 mmHg] and diastolic [87.7+9.6 vs 83.9+7.8 mmHg]), higher glycated hemoglobin (HbA1c) (8.4+1.4 vs 7.6+1 years), higher BMI (28.3+2.8 vs 27.2+2.1 kg/m^2^), presence of chronic kidney disease (CKD) (210 [62.5 %] vs 118 [16.5%]), and a positive family history of cardiac ailments (185 [55.1%] vs 122 [17.1%]) (*p*<0.05 for all). The above factors also correlated with increased chances of S+ for HF on regression analysis.

Conclusion

S+ for HF is common in people with T2DM attending OPDs. The S+ was associated with increasing age, longer duration of T2DM, smoking and tobacco chewing, uncontrolled hypertension and T2DM, obesity, the presence of CKD, use of pioglitazone and insulin, and positive family history. It is the need of the hour to widely extend routine screening for HF in T2DM patients using NT-proBNP in the OPD setting so that benefits of guideline-based therapy can be extended.

## Introduction

Type 2 diabetes mellitus (T2DM) is reaching epidemic proportions across the globe, and the numbers have also skyrocketed in India. As of 2019, India may be home to 87.6 million people with T2DM, with a prevalence of 9.3%. Along with the T2DM burden, the burden of complications of T2DM is also increasing [1].

Heart failure (HF) is an underrecognized complication of T2DM. Once it sets in, it affects the quantity and quality of life of a person with T2DM. HF can also be the first presenting feature of cardiovascular disease in T2DM. Due to the increasing prevalence of T2DM in the society, the prevalence of HF is also expected to go up and can be a major contributor to mortality and morbidity. The prevalence of HF in T2DM is variable, but the American Diabetes Association (ADA) position statement estimates it to be close to 22%. Both diabetes and prediabetes increase risk of HF almost two to four times. With the availability of newer therapies and better understanding of the disease, it is important that HF is recognized before hospitalization and treated so that hospitalization, mortality, and morbidity due to the same can be prevented [1-3].

HF is classified as stage A when there is a presence of risk factors but no symptoms, structural heart disease, or elevated biomarker. Asymptomatic people with structural heart disease or elevated biomarker of myocardial strain are classified as stage B (natriuretic peptides brain natriuretic peptide [BNP] and N-terminal pro-B-type natriuretic peptide [NT-proBNP] are the approved biomarkers with cutoffs of 35 pg/mL and 125 pg/mL, respectively). Diagnosing people in stage B can prevent/delay their progression to symptomatic stages C and D. One of the objective ways to diagnose people in stage 2 has been use of natriuretic peptides, which are endorsed by ADA in their consensus statement in 2022 as well as in standards of medical care for 2024 [2,3].

This clinical practice for screening for HF using natriuretic peptides, especially NT-proBNP, is common in emergency rooms but not yet universally accepted across the globe among asymptomatic T2DM people attending regular outpatient department (OPD) follow-ups. NT-proBNP is preferred over BNP because it is more stable and has a longer half-life of 120 minutes versus 20 minutes for BNP. NT-proBNP also has better sensitivity and predictive value for the diagnosis of HF. It also does not cross-react with BNP [4-6].

The ADA position paper in 2022 endorsed screening for HF among individuals with T2DM with risk factors for HF, and the 2024 ADA guidelines endorsed annual screening for people with T2DM for HF using a biomarker, most notably NT-proBNP. Also, the treatment guidelines for T2DM subclassify treatment of T2DM with HF in a different way (prioritizing sodium-dependent glucose cotransporter 2 [SGLT-2] inhibitors and glucagon-like peptide 1 [GLP-1] receptor agonists over other medications) as compared to those without T2DM. The European Society of Cardiology (ESC) guidelines also endorse the use of NT-proBNP as a screening test for evaluating the risk of HF [2,3,7,8].

NT-proBNP is the most validated marker for screening HF risk, and guideline-directed medical therapy in those who screen positive (S+) has been shown to reduce the risk of hospitalization as well as mortality and morbidity. This increased risk is seen despite normal ECG and 2D echocardiography. High prevalence of HF, as assessed by NT-proBNP, has been documented in previous studies, implying that the need for guideline-directed medical therapies to prevent HF hospitalization and mortality may be needed by a much greater number of people with T2DM as compared to those who are currently getting it [9,10].

The present study conducted at a tertiary care center in India is a retrospective cross-sectional observational study of extending routine NT-proBNP screening among asymptomatic T2DM people attending OPD for consultations with normal ECG and no history of any cardiac ailment. Indian population is known to be at high risk of HF and ischemic heart disease, as well as T2DM. Data on the prevalence of HF in stable Indian T2DM are lacking and can be high considering the vulnerability. The Indian guidelines by the Endocrine Society of India, and the Research Society for Study of Diabetes in India in 2020, and the Research Society for Study of Diabetes in India consensus statement endorse that HF is common in T2DM and merits attention but does not yet incorporate universal screening for HF in the OPD setting [11-15].

## Materials and methods

This retrospective cross-sectional observational study was conducted after approval by the Bhaktivedanta Hospital Ethics Committee for Biomedical Health and Research (protocol no. BMRC/26/2023). A sample size of 264 was considered significant based on the previous reports of prevalence of HF of 22%. At a sample size of 1,049, the margin of error stands at 2.51% [2,16].

Consecutive people with T2DM who attended the OPD of the tertiary care endocrinology and internal medicine departments for consultations and got their NT-proBNP levels screened between August 2022 to July 2023 (practice of advising same started after July 2022 ADA position paper endorsing routine NT-ProBNP screening in T2DM in OPD setting) in the age group of 18-75 years were included in the study. Individuals with known ischemic heart disease or any other known cardiac ailment, abnormal electrocardiogram (ECG), cardiac arrythmias, prior HF, chronic kidney disease (CKD) (eGFR<60 mL/min), chronic liver disease (ALT/AST>2 times the upper limit of normal), respiratory failure, malignancy, pregnancy, recent infection or any terminal illness, and active tuberculosis were excluded from the study. The samples of individuals who were screened for occult unrecognized HF (known T2DM with normal ECG and no known cardiac ailment) were separately labelled as Diab NT-proBNP (both at the level of ordering clinician and laboratory entry). The medical records of these individuals were reviewed by investigating clinicians. All individuals’ age, sex, duration of diabetes, smoking/tobacco chewing status, symptoms if any, and past and family history including those of cardiac ailments were noted. Details of clinical examination, anthropometry including height, weight, and body mass index (BMI), pulse rate, and blood pressure recording were also noted. Investigations including glycated hemoglobin (HbA1C), lipid profile, renal function test, and liver function test conducted in the last one month were recorded from the hospital clinical and laboratory records. Details of ongoing medications and underlying ailments if any were also recorded. Samples were collected at the time of their blood collection for other routine tests in the fasting state, and NT-proBNP was tested on the same day using an electrochemiluminescence (ECLIA) assay kit (Elecsys proBNP II, Roche Diagnostics, Mannheim, Germany). A cutoff of >125 pg/mL was taken as significant for diagnosis of stage B of HF (S+ for HF). Those with levels <125 pg/mL were labelled as screen negative (S-) for HF.

Individuals who were found to be S+ for HF underwent evaluation by a cardiologist (being referred to the cardiac department as standard of care) including 2D echocardiography and, if deemed necessary, stress test or coronary calcium scoring or coronary angiography. Also, therapeutic changes were made as per guidelines, and instructions for follow-up were given.

Statistical analysis

Data analysis was conducted using the R programming language version 4.2.3 (R Foundation for Statistical Computing, Vienna, Austria). The results are summarized as frequency and percent for nominal variables and as mean (SD) for numerical variables. We classified NT-proBNP level above 125 ng/mL as S+ for HF. Differences in distribution in numerical variables between those who screened positive compared to others were tested using the Mann-Whitney U test, while chi-squared tests were used for nominal variables. We used logistic regression analysis to find out independent factors associated with HF. A p-value of <0.05 was considered statistically significant. For measuring the differences in the two percentages (proportions), we used the N1 chi-squared test by Cambell and Richardson.

## Results

A total of 1,049 people with T2DM in the age range of 18-75 years (mean age 56.9+10.9) were screened for HF in the study. Of these, 574 (54.7%) were females. Their general characteristics and laboratory values are given in Table 1. Of these, 336 (32.03%) had an NT-proBNP level above 125 ng/mL, confirming S+ for HF. The median age for the positive group was significantly higher at 63.5 years compared to 55 years in the S- group (p<0.001). The S+ for HF was similar among females, 175/574 (31.18%), versus males, 157/475 (33.08%) (p=0.519). Duration of diabetes was longer in the S+ group (median 12 years) than in the S- group (median 8 years; p<0.001). Smoking prevalence was notably higher among S+, 94 (28.0%), compared to S-, 55 (7.7%) (p<0.001). Tobacco chewing was also more common among those who were S+, 66 (19.6%), than among those who were S-, 24 (3.4%) (p<0.001). Heart rate was higher among those who were S+ as compared to those who were S-. Hypertension was more common in the S+ group, 310 (92.3%), than in the S- group, 481 (67.5%) (p<0.001). Blood pressure, both systolic and diastolic, was significantly higher in the S+ group (p<0.001). The median number of blood pressure medications was also higher in the S+ group (3.0) compared to the S- group (2.0) (p<0.001). Total cholesterol levels, low-density lipoprotein, BMI, HbA1c, and the number of diabetes medications showed significant differences, with higher values in the positive group (all p<0.001). Family history of cardiovascular diseases was more common among those who were S+, 185 (55.1%), versus those who were S-, 122 (17.1%). People with CKD and T2DM were more likely to be S+ for HF, 210 (62.5%), than those without CKD, 118 (16.5%).

**Table 1:** Distribution of characteristics across those who screened positive and and those who screened negative for HF ^1^Data are presented as mean (SD) or n (%) ^2^Wilcoxon rank sum test, Pearson's chi-squared test, or Fisher's exact test BP, blood pressure; CHO cholesterol; HDL, high-density lipoprotein; LDL, low-density lipoprotein; BMI, body mass index; HbA1c, glycated hemoglobin; DM, diabetes mellitus; CKD, chronic kidney disease; NT-proBNP, N-terminal pro-B-type natriuretic peptide

Characteristic	Overall (N=1,049)^1^	Negative (N=713)^1^	Positive (N=336)^1^	t-Value/Chi-squared value	p-Value^2^
Age	56.9 (10.9)	54.2 (10.6)	62.5 (9.3)	-12.3	<0.01
Female	574 (54.7%)	395 (55.4%)	179 (53.3%)	0.416	0.519
Diabetes duration	11.1 (7.1)	9.6 (6.1)	14.4 (7.8)	-10.841	<0.01
Smoking	149 (14.2%)	55 (7.7%)	94 (28%)	171.24	<0.01
Tobacco chewing	90 (8.6%)	24 (3.4%)	66 (19.6%)	75.079	<0.01
Hypertension	791 (75.4%)	481 (67.5%)	310 (92.3%)	74.409	<0.01
Number of BP medicines	1.9 (1.2)	1.5 (1.2)	2.6 (1.1)	-14.22	<0.01
Heart rate	79.8 (11.6)	76.6 (9.4)	86.6 (12.8)	-14.24	<0.01
CHO	165.4 (34.6)	159.9 (30.1)	177.3 (40.3)	-7.80	<0.01
HDL	39.2 (7.8)	39.2 (7.7)	39.2 (8.0)	0	0.873
LDL	88.8 (28.9)	84.3 (25.6)	98.3 (33.0)	-7.50	<0.01
BMI	27.5 (2.4)	27.2 (2.1)	28.3 (2.8)	-7.08	<0.01
HbA1c	7.9 (1.2)	7.6 (1.0)	8.4 (1.4)	-10.57	<0.01
Number of DM medications	2.8 (0.6)	2.7 (0.6)	3.1 (0.6)	-10.07	<0.01
Family history	307 (29.3%)	122 (17.1%)	185 (55.1%)	157.04	<0.01
CKD	328 (31.3%)	118 (16.5%)	210 (62.5%)	222.24	<0.01
NT-proBNP	172.1 (466.1)	42.6 (26.7)	447.1 (752.8)	-14.34	<0.01
Systolic BP	140.2 (18.6)	134.6 (15.0)	152.1 (19.9)	-15.81	<0.01
Diastolic BP	85.1 (8.6)	83.9 (7.8)	87.7 (9.6)	-6.82	<0.01

We performed logistic regression analysis, whose results are tabulated in Table 2. The primary outcome was an NT-proBNP level greater than 125 pg/mL, indicative of S+ for HF among 1,049 individuals. The model included age, diabetes duration, smoking status, hypertension, heart rate, BMI, HbA1c levels, CKD, and family history as predictors. The deviance residuals ranged from -2.3215 to 2.9542, suggesting variability in prediction accuracy across the dataset with some notable outliers. The substantial reduction in residual deviance to 660.96 from a null deviance of 1301.87 on comparable degrees of freedom highlights the enhanced fit provided by the inclusion of these predictors. The Akaike Information Criterion (AIC) for the model stood at 682.96, suggesting a reasonable trade-off between model complexity and fit. Notably, the model converged efficiently within six iterations of Fisher scoring. The R² Tjur value of 0.551 indicates that the model explains approximately 55.1% of the variability in the NT-proBNP levels, a substantial explanatory power given the complexity of factors influencing HF. The analysis revealed several key factors significantly associated with an elevated risk of HF. Age demonstrated a consistent association, with a slight decrease in odds ratio (OR) from 1.09 (95% CI: 1.07-1.11) on univariable analysis to 1.07 (95% CI: 1.04-1.10) on multivariable analysis, indicating the influence of other variables. The duration of diabetes was initially significant on univariable analysis (OR: 1.10, 95% CI: 1.08-1.13), but this association was attenuated upon adjustment for other factors. Notably, smoking emerged as a strong predictor, with oral tobacco users and current smokers showing markedly elevated risks in both univariable and multivariable models. Hypertension was another significant predictor, though its effect size was reduced in the multivariable model. Heart rate and BMI were consistently associated with higher odds of HF. The analysis also highlighted the importance of HbA1c levels, family history of cardiovascular diseases, and the presence CKD as significant factors. These findings underline the multifactorial nature of HF risk and emphasize the need for comprehensive risk assessments incorporating these identified factors.

**Table 2:** Logistic regression analysis of variables with their odds ratio and correlation with screen positivity for heart rate BP, blood pressure; HbA1c, glycated hemoglobin

	Univariate regression			Multivariate regression		
Characteristic	OR	95% CI	p-Value	OR	95% CI	p-Value
Age	1.09	1.07, 1.11	<0.001	1.07	1.04, 1.10	<0.001
Sex (female/male)	1.09	0.84,1.41	0.519			
Diabetes duration	1.10	1.08, 1.13	<0.001	1.02	0.99, 1.06	0.228
Smoking	6.16	4.26, 8.98	<0.01	5.21	3.03,9.09,	<0.01
Tobacco chewing	9.91	6.11, 16.6	<0.01	12.7	6.59, 25.5	<0.01
Hypertension	5.75	3.81, 9.03	<0.01	1.85	1.04, 3.36	< 0.01
Number of BP medicines	2.59	2.22, 3.04	<0.01			
Dyslipidemia	1.18	0.72, 1.99	0.51			
Heart rate	1.09	1.08, 1.11	<0.001	1.07	1.05, 1.09	<0.001
Body mass index	1.20	1.14, 1.27	<0.001	1.16	1.07, 1.26	<0.001
HbA1c	1.67	1.49, 1.88	<0.001	1.36	1.16, 1.61	<0.001
Number of diabetes medicines	2.70	2.12, 3.47	<0.001			
Family history	5.94	4.45, 7.95	< 0.01	6.73	4.43, 10.4	<0.01
Chronic kidney disease	8.40	6.27, 11.3	<0.01	4.81	3.21, 7.27	<0.01

 Among those who were S+ for HF, the use of pioglitazone and insulin was more and use of SGLT-2 inhibitors was less. There was no difference in use of other anti-diabetic medications among the two groups. The details of differences in anti-diabetic medication use among the two groups are shown in Table 3.

**Table 3:** Differences in anti-diabetic medication use among those who screened positive for HF versus those who screened negative for HF. DPP-4, dipeptidyl peptidase 4; SGLT-2, sodium-dependent glucose cotransporter 2; GLP-1, glucagon-like peptide 1; HF, heart failure

	Screen positive (N=336)	Screen negative (N=713)	Chi-squared value	p-Value
Metformin	320 (95.2)	680 (95.4)	0.004	0.92
Sulphonylureas	226 (67.3)	468 (65.6)	0.201	0.59
DPP-4 inhibitors	265 (78.9)	554 (77.7)	1.517	0.64
SGLT-2 inhibitors	206 (61.3)	498 (69.9)	7.158	<0.01
GLP1-receptor agonists	16 (4.8)	38 (5.3)	0.056	0.76
Glitazones	26 (7.7)	6 (0.8)	34.43	<0.01
Glitazar	30 (8.9)	62 (8.7)	0.001	0.90
Alpha glucosidase inhibitors	74 (22)	148 (20.8)	0.150	0.63
Insulin	102 (30.3)	164 (23)	6.146	0.01

**Figure 1:**
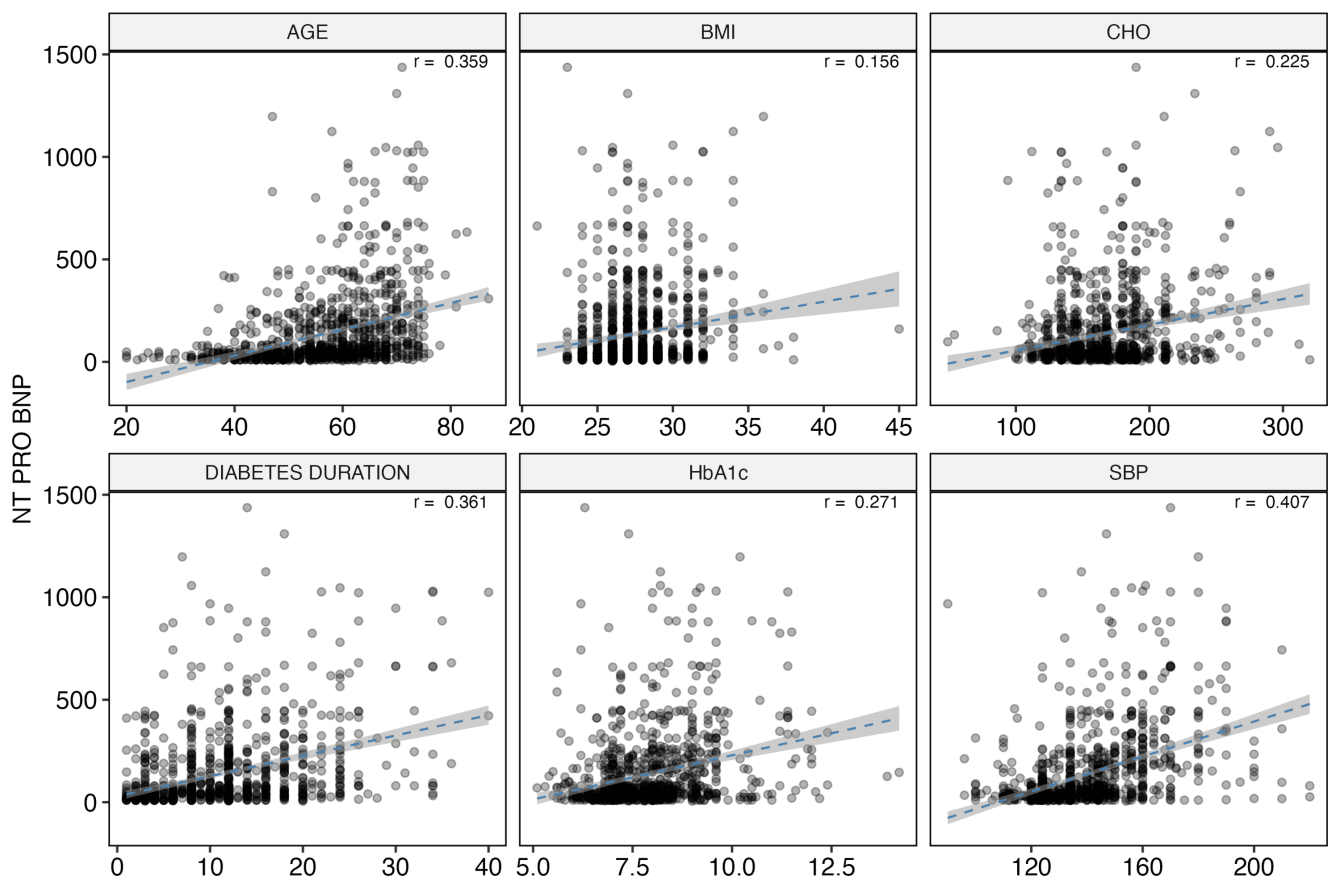
Correlation of NT-proBNP with age (in years), body mass index (in kg/m2), total cholesterol (mg%), diabetes duration (in years), HbA1C (%), and systolic blood pressure (in mmHg) (p<0.05 for all) NT-proBNP, N-terminal pro-B-type natriuretic peptide; HbA1c, glycated hemoglobin

The NT-proBNP levels have been used as a marker of HF, and serial measurements have been used to judge the utility of interventions for prevention of HF hospitalization. Many variables were identified as risk factors for S+ for HF using NT-proBNP. Continuous variables exhibited significant correlation with NT-proBNP values (p<0.05). The correlation of NT-proBNP with age, duration of diabetes, BMI, HbA1C, cholesterol levels, and systolic blood pressure SBP is shown in Figure 1.

## Discussion

T2DM is a chronic metabolic disorder characterized by hyperglycemia, resulting in microvascular and macrovascular disease. Heart disease is the leading cause of mortality and morbidity in T2DM. HF is an important complication of T2DM, which is often overlooked. HF is caused by multiple contributing factors, including direct damage due to hyperglycemia and advanced glycemic end products, and also contributed by other comorbidities such as hypertension, obesity and dyslipidemia, oxidative stress, and neurohormonal activation (both renin-angiotensin system and sympathoadrenal system). Other risk factors such as family history, advancing age, family history, and genetic susceptibility, and modifiable factors such as smoking and tobacco chewing also contribute to the risk of HF in T2DM. At the structural level, there can be ischemic heart disease as well as cardiomyopathy and microcirculation defects in the coronary circulation, which can contribute to HF. The process evolves over time and has been now classified from stage A (for person with risk factors) to stage B (elevated biomarker) and later stage C and D (evolved HF with structural disease and finally disabling HF) [2,6].

Biomarkers such as NT-proBNP help diagnose HF in occult stage and help initiate therapeutic measures for the prevention of worsening and hospitalization. The current guidelines for the treatment of T2DM demand cardiorenal risk stratification of people with T2DM and then initiation of appropriate therapeutic regimens. High prevalence of HF in T2DM makes it therefore compelling to screen for it routinely in people with T2DM. Though recommended by ADA and other societies, it is not yet a commonly followed practice in OPD catering to people with T2DM. NT-proBNP is the most validated sensitive and specific marker for the diagnosis of HF in chronic care setting, with its use endorsed by ESC and ADA. Its contribution to HF management is enhanced by the fact that it can be measured easily, with treatments based on the same being proven to prevent hospitalization for HF [1,2,6,7,8,11].

The present study is a retrospective cross-sectional observational study of extending routine NT-proBNP screening of people with T2DM attending OPD for regular visit at a tertiary care center in India. Out of 1,049 people with T2DM, 336 were S+ for HF, amounting to a prevalence of 32.03% in the population. T2DM is a major risk factor for HF, and a high incidence of T2DM in people with HF has been reported in the past. However, our study is the first from India to report a high prevalence of HF among T2DM people attending OPD who were screened as per the ESC and ADA guidelines. This is similar to that reported by Ceriello et al. and higher than reported by Rajput et al. The study by Rajput et al. used a BNP level of 105 pg/mL and reported HF prevalence as 10%. This cutoff is much higher than the cutoff of 35 pg/mL recommended by ESC guidelines. Indian guidelines also endorse the high prevalence of HF in T2DM as well as the relevance of diagnosing the same in the treatment of T2DM but do not recommend universal screening for HF in T2DM using a biomarker as of date, and our study makes a case for universal screening or at least risk stratification and screening in high risk [3,8,10,13-15].

Prevalence of occult HF was almost equal among males as compared to females, The prevalence of S+ for HF was similar among males and females, which is similar to that reported by Ceriello et al. as well as reported in the literature before. Some studies have reported males being affected more than females due to the high prevalence of coronary artery disease among them, but HF with preserved ejection was common in females. Our study reported almost equal prevalence in both genders, and in majority of cases, the HF was with preserved ejection fraction, which was detected early and without hospitalization [13,17,18].

Smoking has been associated with increased NT-proBNP levels as well as risk of HF in previous studies, and stopping smoking is a part of cardiac rehabilitation measures. Our study also linked smoking with HF. Tobacco chewing is a common practice in India and was also strongly associated with the risk of HF. The association was stronger for tobacco chewing as compared to smoking. Hence, it is important to discourage tobacco chewing especially in people with T2DM [19].

Duration of diabetes and age are important risk factors for HF. HF in the elderly is proposed to be due to aging, with hypertension, T2DM, and obesity proposed as possible risk enhancers for myocardial aging. In our study, all individuals had T2DM, and the longer the duration of DM, the more the risk for S+ for HF. Also, the average age of those who were S+ for HF was more than those who were S- for HF, underlining the fact that the longer the duration or T2DM and the higher the age, the higher the risk of HF. Myocardial aging is accelerated by the presence of T2DM. Hypertension is an important risk factor for HF. Hypertension, which is common in T2DM, also accelerates myocardial aging. Also, hypertension drives cardiac remodeling of the left ventricle and increases the risk of HF. HF resulting in increased sympathetic drive also increases heart rate and blood pressure, which is reflected in our observations of higher systolic and diastolic blood pressures as well as heart rates in people who were S+ for HF than those who were S- [20-22].

Obesity is a known risk factor for HF, and higher BMI was more commonly associated with the risk of S+ for HF. Our study confirmed the past observation that we are currently in a dual pandemic of obesity and HF. Obesity is rising to pandemic proportions and is more common in individuals with socioeconomic disadvantage whose number is higher in India. Diabetes and obesity, commonly referred to as diabesity, also compound the risk of HF, and hence T2DM with obesity warrants regular screening for HF, and NT-proBNP can be a useful tool for the same [23,24].

Glycemic control is an important risk factor for HF. Good glycemic control (HbA1c<7%) should be the aim in most individuals with T2DM. Poor glycemic control (HbA1C>8%) increases risk of HF. The mean HbA1c levels were above 8% in both groups (S+ as well as S-), but the HbA1c levels were higher in those who were S+ as compared to those who were S-, and the HbA1C levels correlated with the risk of S+ for HF, implying relevance of glycemic control in the prevention of cardiac risk, as pointed out by previous studies. The number of T2DM medications as well as hypertension medication need was more in people with S+ for HF as compared to those who were S-, which can be explained by the longer duration of T2DM as well as more prevalence of hypertension among those who were S+ as compared to those who were S- [25,26].

S+ for HF was much more common in people with T2DM who had CKD as compared to those who did not have CKD, emphasizing that CKD is a strong risk factor for HF. Since people with T2DM with eGFR below 60 were excluded from the study, main bulk of the population had albuminuric CKD with normal or mildly deranged serum creatinine levels, and this strengthens the fact that albuminuria can be a risk factor for HF. It goes beyond saying that the presence of albuminuria also adds increased incidence of hypertension, which further increases the risk for risk [27].

Family history as a risk factor is missed in most risk engines. Family history of heart diseases including ischemic heart disease is an underrecognized risk factor for HF, and our study highlights the need to emphasize the same. It has been felt that South East Asians including Indians are vulnerable to early onset heart disease, and it can occur earlier in subsequent generations, and hence there is a need to look at family history of heart disease as an important risk factor for HF [28].

While pioglitazone use is a known risk factor for HF, the difference in the two groups as regards insulin use may also be due to the duration of diabetes being longer in the S+ group, resulting in poor beta cell reserve and need for insulin. SGLT-2 inhibitor use reduced the chances of S+ for HF, as has been noted in most of the cardiovascular outcome trials [29].

Those who were S+ were further evaluated whenever possible. A 2D echocardiography was available for 262 of the 336 people, and the commonest abnormalities seen were diastolic dysfunction, 192 (73.2%). Ejection fraction was preserved (>50%) in 250/262 (95.4%). Regional wall motion abnormalities were seen in 26 (9.92%). There was heterogenicity in reporting of 2D echocardiography findings across people who underwent same, and hence there is a need for an objective and sensitive modality such as NT-proBNP for early diagnosis of HF in T2DM. Coronary calcium scoring records were available for 194 people who were S+, and 131/194 (71.3%) had coronary calcium scores above 100, indicating ischemic heart disease being an important contributor to S+ for HF.

Limitations and bias of the study

Since this is a retrospective cross-sectional study conducted on the OPD population at a tertiary care center, the data may not be applicable to the general population. The study predominantly looks at screen positivity of a biomarker and not actual HF hospitalization. There is possibility of some false positives due to conditions such as respiratory infections, though the investigators have been excluding screening for biomarker in most of the conditions that can falsely elevate NT-proBNP. Since this is a retrospective cross-sectional observational study for biomarker positivity, further evaluation details and follow-up are not available of each person who was S+.

The study was conducted at a tertiary care center in a metro city, and thus there can be referral bias with more complicated people being referred to the institute. Since this is also a real-world retrospective cross-sectional observational study of extending the routine NT-proBNP screening in people with T2DM coming for regular follow up, there is a possibility that people who had more nonspecific symptoms such as fatigue opted to get tested.

Strengths of the study

The present study highlights the high prevalence of occult undiagnosed HF in the Indian population, which is traditionally considered vulnerable to HF and ischemic heart disease. It highlights the need to pursue more aggressive screening for HF among asymptomatic T2DM people during their routine OPD visits using NT-proBNP to initiate remedial measures to prevent hospitalization due to HF. This is also the first real-world retrospective cross-sectional observational study of routine NT-proBNP screening in asymptomatic OPD people with T2DM and gives a glimpse that occult undiagnosed HF is common and needs routine screening for risk stratification and streamlining care of people with T2DM according to guidelines. It also gives clinicians an idea about which population subgroups are at high risk of S+, and thus screening for HF can be more proactively pursued in these subgroups. Our study apart from highlighting known risk factors for HF emphasizes the need to consider tobacco chewing and positive family history of heart disease as risk factors for HF [9,30].

The following recommendations can be derived from the study: 1) routine screening of undiagnosed HF by biomarker NT-proBNP in people with T2DM with normal ECG is important and may be followed in OPD practice, 2) stopping smoking, improving glycemic and blood pressure control, weight reduction, and HF-friendly therapeutics can reduce the risk of HF, 3) the residual risk can still be contributed by aging, duration of T2DM, and family history of heart disease, and 4) a prospective study that is sponsored (so as to eliminate bias of choice to conduct the test from out-of-pocket expenses) and conducted at a population level can give a better idea about the true prevalence of HF in the population.

## Conclusions

S+ for HF is common in people with T2DM attending the OPD. The S+ was associated with increasing age, longer duration of T2DM, smoking and tobacco chewing, uncontrolled hypertension and T2DM, obesity, presence of CKD, and positive family history. It is the need of the hour to widely extend routine screening for HF in T2DM using NT-proBNP in the OPD setting so that the benefits of guideline-based therapy can be extended to the needy.
